# Musculoskeletal Symptoms, Upper-Limb Disability and Burnout Among Hairdressers Working in Women’s Hairdressing Salons in Istanbul, Turkey: A Cross-Sectional Study

**DOI:** 10.3390/healthcare14213815

**Published:** 2026-11-09

**Authors:** Adem Çalı, Haniyeh Moghani, Zahra Moghani, Bita Tavakkoli Kariznowee, Mersedeh Pazoki, Ömer Şevgin

**Affiliations:** 1 Department of Physiotherapy and Rehabilitation, Faculty of Health Sciences, İstanbul Topkapı University, 34087 Istanbul, Turkey; 2 Department of Physiotherapy and Rehabilitation, Faculty of Health Sciences, Üsküdar University, 34768 Istanbul, Turkey; hanieh.moghani@st.uskudar.edu.tr (H.M.); zahra.moghani@st.uskudar.edu.tr (Z.M.); bita.tavakkolikariznowee@st.uskudar.edu.tr (B.T.K.); mersedeh.pazoki@st.uskudar.edu.tr (M.P.); omer.sevgin@uskudar.edu.tr (Ö.Ş.)

**Keywords:** hairdressers, occupational musculoskeletal disorders, Nordic questionnaire, QuickDASH, burnout, ergonomics

## Abstract

**Background:** Hairdressing combines sustained arm elevation, repetitive precision grip, prolonged standing and high emotional labour, yet the joint contribution of physical and psychosocial load to musculoskeletal outcomes in this workforce is rarely quantified. We determined the twelve-month prevalence of musculoskeletal symptoms, upper-limb disability and burnout among hairdressers working in women’s hairdressing salons in Istanbul, Turkey, and identified factors associated with a high symptom burden. **Methods:** In this cross-sectional study, 100 hairdressers registered with the Istanbul Chamber of Women’s Hairdressers and Manicurists were assessed with the Standardised Nordic Questionnaire (Turkish version), the QuickDASH (Turkish version) and the Maslach Burnout Inventory (Turkish adaptation; emotional exhaustion, depersonalisation, personal accomplishment subscales). Participants were dichotomised a priori into low (≤2 affected regions, *n* = 43) and high (≥3 regions, *n* = 57) symptom-burden groups. Analyses included Spearman correlations, t/Mann–Whitney U tests (with Holm correction as a sensitivity analysis), multiple linear regression, univariable/multivariable logistic regression constrained by the events-per-variable rule, and sensitivity analyses modelling the continuous number of affected regions (Poisson regression with robust standard errors). **Results:** Participants (63 men and 37 women; mean age 35.3 ± 6.5 years; mean tenure 12.6 ± 5.8 years) reported symptoms in at least one region in 95% of cases (95% CI 88.7–98.4). Prevalence was highest for the neck (48%), upper back (44%), shoulder (41%), and lower back (38%). Mean QuickDASH was 30.5 ± 9.7, but it was not associated with regional symptom burden. The high-burden group reported higher emotional exhaustion (*p* = 0.029) and depersonalisation (*p* = 0.040), lower personal accomplishment (*p* = 0.014), and greater pain frequency (*p* = 0.002) and intensity (*p* < 0.001); after Holm correction, only the pain measures remained significant. In the multivariable model restricted to sociodemographic, occupational, and burnout variables, shorter occupational tenure remained associated with high burden (adjusted OR 0.92 per year, 95% CI 0.85–0.99; AUC = 0.714). In sensitivity analyses treating the number of affected regions as a continuous count, personal accomplishment was the only burnout dimension independently associated with symptom burden (incidence rate ratio 0.965, 95% CI 0.943–0.987), whereas the tenure association attenuated (*p* = 0.071). **Conclusions:** Musculoskeletal symptoms were near-universal in this sample and clustered in the neck–shoulder–upper back complex. All three burnout dimensions were worse in high-burden workers, with reduced personal accomplishment the most robust correlate of symptom burden, and workers earlier in their careers carried a higher multi-site burden—cross-sectional associations that are compatible with, but do not demonstrate, a healthy-worker survivor effect. These hypothesis-generating findings require confirmation in prospective, multicentre studies with objective exposure assessment and comparison groups. Preventive programmes should pair ergonomic redesign and exercise with measures that protect professional efficacy.

## 1. Introduction

Work-related musculoskeletal disorders (WMSDs) remain the leading cause of occupational disability worldwide, accounting for a substantial share of sickness absence, productivity loss and premature exit from the labour market [1]. Low back pain alone affected an estimated 619 million people in 2020 and remains the single largest cause of years lived with disability, with occupational ergonomic factors among its principal attributable risks [2,3]; neck pain adds a further 203 million prevalent cases globally [4]. According to the WHO/ILO joint estimates, occupational exposure to ergonomic risk factors—including awkward and static postures, repetitive movement, forceful exertion and prolonged standing—is among the most widespread occupational exposures worldwide [5]. WMSDs are typically multifactorial: repetitive movement, forceful exertion, sustained non-neutral postures, insufficient recovery, and adverse psychosocial conditions each contribute and frequently interact [1,6].

Hairdressing concentrates several of these exposures with unusual intensity. A scoping review of the occupational-health literature in hairdressing identified six recurring exposure categories: strenuous upper-limb postures with the arms abducted or elevated, awkward spinal positioning with forward flexion and rotation, high workload with insufficient breaks, prolonged standing, mental stress, and specific task demands such as cutting, colouring and blow-drying [6]. Work above shoulder level is an established risk factor for shoulder complaints [7]. Prolonged standing predisposes to low back pain [8] and lower-limb symptoms [9], and reported twelve-month prevalence estimates in hairdressers vary widely across settings—roughly 13–76% for the lower back, 9–58% for the neck and 28–60% for the shoulder [6]. Large studies from Ethiopia and India reported overall WMSD prevalence of 70% and 63%, respectively, with marked between-country differences in the regional pattern [10,11], and further studies from Nigeria, India and Kenya confirm a consistently high burden in salon workers [12,13,14]. Hand and wrist problems are an additional occupational signature of the trade [15].

A second, less-studied feature of hairdressing is its emotional-labour component: continuous client interaction, appearance-related expectations, time pressure and income tied directly to client volume. Burnout—conceptualised by Maslach and Jackson as the triad of emotional exhaustion, depersonalisation and reduced personal accomplishment [16,17]—is therefore plausible in this workforce [18], and emotional labour is a robust antecedent of burnout in customer-facing occupations [19,20]. Longitudinal evidence indicates that workplace psychosocial strain predicts subsequent musculoskeletal morbidity [21,22], and a systematic overview of review-level evidence confirms consistent risk effects of adverse psychosocial work characteristics—high demands, low job control, low social support and effort–reward imbalance—on musculoskeletal disorders, absenteeism and workplace accidents [23]. Cross-sectional studies continue to document the co-occurrence of burnout and musculoskeletal complaints in diverse working populations [24,25]. In Turkey, a recent study of hairdressers reported that low back pain was associated with higher ergonomic risk and occupational burnout [26], but data combining a standardised multi-region symptom survey, a validated upper-limb disability measure and a validated burnout inventory in this workforce remain scarce.

An additional and under-recognised feature of the Turkish setting is the composition of the workforce in women’s hairdressing salons: although the clientele is female, a substantial proportion of the operators are men, so studies framed as investigations of “female hairdressers” may misrepresent the population actually at risk. The present study therefore sampled hairdressers working in women’s salons irrespective of the operator’s own sex.

The aims of this study were: (i) to determine the twelve-month prevalence of musculoskeletal symptoms by body region in hairdressers working in women’s hairdressing salons in Istanbul; (ii) to quantify upper-limb disability and burnout in this group; and (iii) to compare workers with a low (≤2 affected regions) and high (≥3 affected regions) symptom burden and identify the sociodemographic, occupational and psychosocial factors independently associated with high burden.

## 2. Materials and Methods

### 2.1. Study Design and Setting

This observational, descriptive cross-sectional study was conducted in Istanbul, Turkey. Participants were recruited from salons affiliated with the Istanbul Chamber of Women’s Hairdressers and Manicurists (İstanbul Kadın Kuaförleri ve Manikürcüleri Esnaf ve Sanatkarlar Odası). Using the Chamber’s registry of affiliated salons, members of the research team visited salons in person, informed salon owners and staff about the study, and invited eligible hairdressers to participate; no e-mail, newsletter, or printed announcement was used. Data were collected between March and August 2026. The study is reported in accordance with the STROBE statement for cross-sectional studies [27].

### 2.2. Participants

Eligible participants were hairdressers of working age (observed range—23–50 years) employed in salons affiliated with the Chamber, actively practising the profession for five years or longer, and literate in Turkish. Individuals with fewer than five years in the profession and those holding a second concurrent job were excluded so that reported symptoms could be attributed to a single, sustained occupational exposure. Recruitment proceeded by consecutive enrolment of volunteers during the salon visits until the target sample of 100 was reached. No formal a priori sample-size calculation was performed. The target of 100 participants was specified before data collection on combined feasibility and analytic grounds: it represented the number of eligible volunteers realistically attainable from the Chamber’s registry within the study window, and it was expected to support a multivariable logistic model with up to four covariates under the events-per-variable rule (EPV ≥ 10; Section 2.4), assuming an approximately balanced split between symptom-burden groups. Post hoc, the achieved sample provides a two-sided 95% confidence interval no wider than approximately ± 10 percentage points for a prevalence of 50%, and the observed 43 non-events permitted exactly four covariates (EPV = 10.8). The absence of a formal power calculation and the limited precision of some estimates are acknowledged in the Limitations.

All participants received verbal and written information about the study and provided written informed consent. The study was conducted in accordance with the Declaration of Helsinki and approved by the Üsküdar University Non-Interventional (Non-Clinical) Research Ethics Committee (protocol code 61351342/020-1611; decision of meeting no. 12, dated 26 November 2025).

### 2.3. Measures

Sociodemographic and occupational form. A purpose-designed form recorded sex, age, height, weight, marital status, educational attainment, years of occupational experience, and mean daily working hours. Body mass index (BMI) was calculated as weight (kg) divided by height squared (m^2^). Participants additionally rated the frequency of work-related pain on a five-point ordinal scale (from 0 = never to 4 = always) and its intensity on a six-point numerical rating scale (from 0 = no pain to 5 = worst imaginable pain). The form did not record whether the participant was an employee or an owner/operator of the salon; the implications of this omission, particularly for the burnout findings, are considered in the Limitations.

Standardised Nordic Questionnaire (SNQ). The SNQ identifies self-reported musculoskeletal trouble (ache, pain or discomfort) during the preceding twelve months in nine anatomical regions: neck, shoulders, upper back, elbows, hands/wrists, lower back, hips/thighs, knees and ankles/feet [28]. A culturally adapted Turkish version, which has demonstrated acceptable reliability and validity, was used [29]. Responses were dichotomous (present/absent). For each participant, the number of symptomatic regions (0–9) was summed as an index of overall symptom burden. Following the a priori analysis plan, participants were classified into a low-burden group (≤2 affected regions) and a high-burden group (≥3 affected regions). This cut-off was specified before analysis on substantive grounds: pain involving several anatomical sites is an established multi-site pain phenotype associated with greater disability and work impairment than localised pain [30], and a threshold of three regions distinguishes workers whose symptoms extend beyond a single functional complex (e.g., the neck–shoulder region) from those with localised complaints. The cut-off subsequently proved to coincide with the sample median (3 regions), yielding well-balanced groups. To ensure that no conclusion depended on this categorisation, key analyses were repeated with the number of affected regions treated as a continuous outcome (Section 2.4).

QuickDASH. The QuickDASH is an abbreviated, 11-item version of the Disabilities of the Arm, Shoulder and Hand (DASH) questionnaire that measures symptoms and physical function of the upper limb as a single unit [31]. Eight items address the degree of difficulty experienced during the preceding week in performing everyday activities (opening a tight jar; performing heavy household chores; carrying a shopping bag or briefcase; washing one’s back; using a knife to cut food; recreational activities involving arm force or impact) and the extent of interference with social activities and with work or other regular daily activities, and three items address symptom severity (arm, shoulder or hand pain; tingling; sleep difficulty due to pain). Each item is scored from 1 (no difficulty/none) to 5 (unable/extreme); provided at least 10 of the 11 items are answered, the score was computed as ((mean item score − 1) × 25), yielding a 0–100 scale with higher values indicating greater disability [31]. The Turkish version has demonstrated acceptable validity and reliability (Cronbach’s α 0.84–0.94) [32].

Maslach Burnout Inventory (MBI). Burnout was assessed with the MBI [16] in its Turkish adaptation by Ergin [33]. In this version, each item is rated on a five-point frequency scale scored 0–4, yielding subscale ranges of 0–36 for emotional exhaustion (9 items), 0–20 for depersonalisation (5 items) and 0–32 for personal accomplishment (8 items). Higher emotional exhaustion and depersonalisation scores and lower personal accomplishment scores indicate greater burnout. Because no consensus clinical cut-off values exist for the Turkish adaptation, the subscales were analysed as continuous variables, in line with current measurement guidance [17,34]; a summed total is reported descriptively only, since the subscales are not unidirectional.

### 2.4. Statistical Analysis

Analyses were performed in Python 3.11 (pandas, SciPy, statsmodels). Continuous variables are presented as mean ± standard deviation (SD) and median with interquartile range (IQR), and categorical variables are presented as frequency and percentage. Normality was assessed with the Shapiro–Wilk test. Exact (Clopper–Pearson) 95% confidence intervals (CI) were calculated for prevalence estimates.

Bivariate associations were examined with Spearman’s rank correlation, since several key variables (occupational tenure, daily working hours, depersonalisation, pain frequency, pain intensity, and number of affected regions) deviated from normality. Burden groups (≤2 vs. ≥3 regions) were compared with the independent-samples *t*-test (with Levene’s test for variance homogeneity) when both group distributions were normal, and with the Mann–Whitney U test otherwise; effect sizes are reported as Cohen’s d or r = Z/√*n*, respectively. Categorical comparisons used the chi-square test (with continuity correction for 2 × 2 tables) or Fisher’s exact test when expected counts were below five. Comparisons of scores between participants with and without symptoms in each region used the Mann–Whitney U test. Because the between-group comparisons involve ten continuous variables, this set was treated as one family of tests; as a sensitivity analysis, Holm’s sequentially rejective procedure was applied across the family, and the Results distinguish associations robust to this correction from those that should be regarded as exploratory.

Multiple linear regression modelled QuickDASH. Multicollinearity was assessed with variance inflation factors (VIFs), residual normality with the Shapiro–Wilk test, homoscedasticity with the Breusch–Pagan test, and independence with the Durbin–Watson statistic; a parsimonious model was obtained by backward elimination at *p* < 0.05. Univariable logistic regression was performed for high symptom burden (≥3 regions), and variables at *p* < 0.20 were eligible for the multivariable model, entered in ascending order of univariable *p* value up to a maximum set by the events-per-variable rule (EPV ≥ 10; 43 non-events → up to four covariates) [35,36]. Model performance was assessed with the likelihood-ratio test, Nagelkerke R^2^, the Hosmer–Lemeshow test, and the area under the receiver operating characteristic curve (AUC) [36]. Odds ratios (ORs) are reported with 95% CIs; two-sided *p* < 0.05 was considered significant.

Because self-rated pain frequency and intensity overlap conceptually with the musculoskeletal outcomes they would predict, all regression models were fitted twice: a primary specification restricted to sociodemographic, occupational and burnout variables, and a secondary specification additionally including the two pain-characteristic variables. Both are reported so that readers can judge the influence of this overlap. Finally, to avoid reliance on any single categorisation of symptom burden, the number of affected regions (0–9) was additionally analysed as a continuous outcome in sensitivity analyses, using Poisson regression with heteroscedasticity-robust (HC1) standard errors (the count showed no overdispersion: Pearson χ^2^/df = 0.67) and the same covariate sets as the logistic models; associations are expressed as incidence rate ratios (IRRs) with 95% CIs.

## 3. Results

### 3.1. Participant Characteristics

One hundred hairdressers were enrolled; there were no missing data. Sixty-three (63.0%) were men and 37 (37.0%) were women, reflecting the workforce composition of women’s hairdressing salons in Istanbul, where male operators predominate. The sociodemographic and occupational characteristics of the sample are summarised in Table 1 and are not repeated in the text.

### 3.2. Prevalence of Musculoskeletal Symptoms

**Figure 1:**
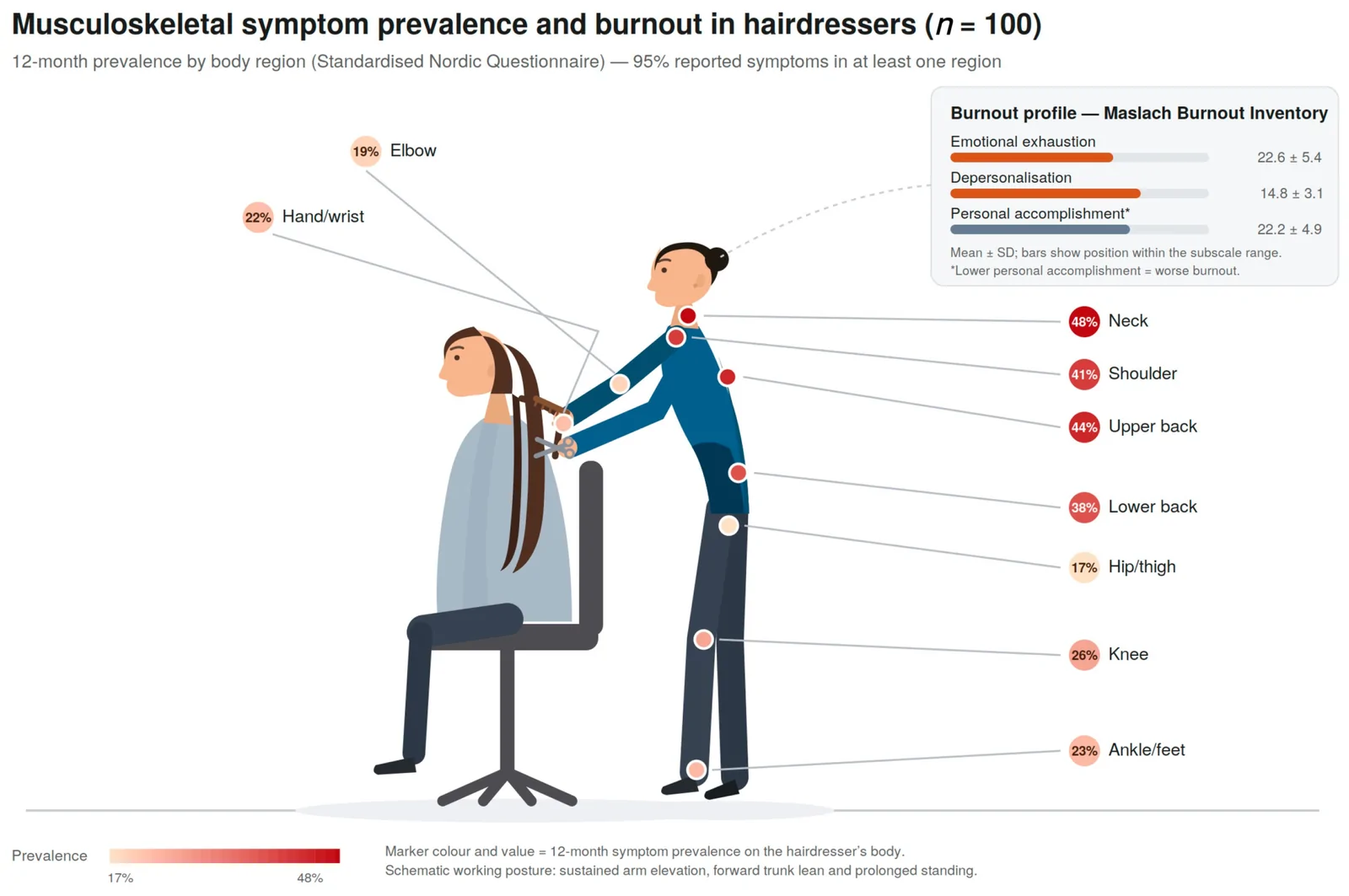
Body map of twelve-month regional symptom prevalence during a typical cutting task, with the burnout profile of the sample (Maslach Burnout Inventory, Turkish adaptation, 0–4 scoring). Marker colour scales with prevalence.

Ninety-five participants (95.0%; 95% CI 88.7–98.4) reported musculoskeletal trouble in at least one body region during the preceding twelve months, and 57 (57.0%) reported symptoms in three or more regions, forming the high-burden group. Symptoms clustered in the axial and proximal upper-limb regions: the neck was the most frequently affected site, followed by the upper back, shoulder, and lower back. In total, 82 participants (82.0%) reported symptoms in at least one region of the neck–shoulder–upper back complex. Regional prevalences with exact confidence intervals are presented in Table 2 and illustrated on the body map in Figure 1; the tabulated values are not repeated in the text.

### 3.3. Upper-Limb Disability, Burnout and Pain Characteristics

Upper-limb disability was moderate on average, and work-related pain was reported at least occasionally (frequency rating ≥ 1) by 95 participants. Distributions of the QuickDASH, the three MBI subscales, and the pain ratings are summarised in Table 3.

### 3.4. Correlations

**Figure 2:**
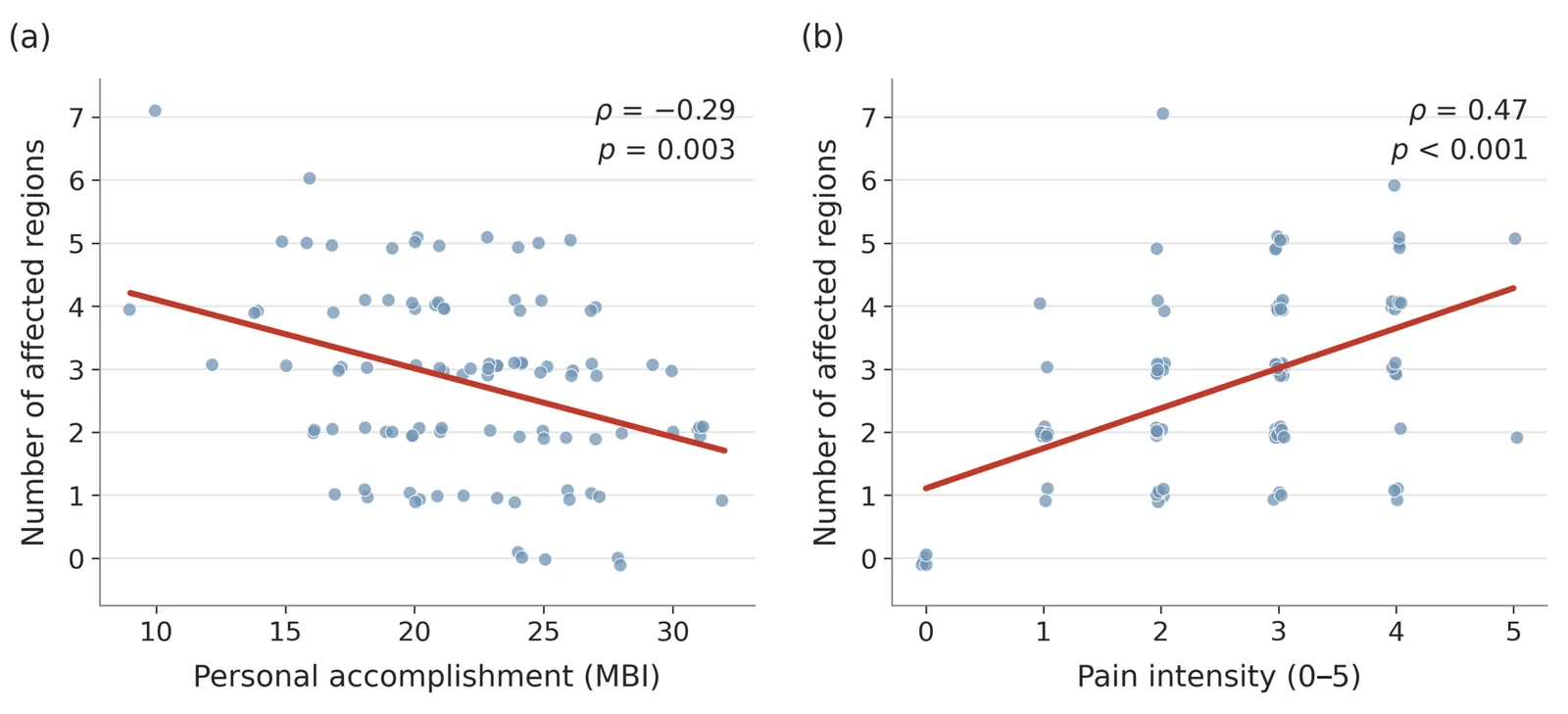
Relationship between the number of affected body regions and (**a**) personal accomplishment (Maslach Burnout Inventory) and (**b**) pain intensity. Solid lines are least-squares fits; *ρ* values are Spearman coefficients; points are jittered for visibility.

The number of affected regions correlated positively with pain intensity and pain frequency, supporting the internal coherence of the symptom measures. Among the burnout dimensions, personal accomplishment showed the most consistent pattern, correlating inversely with the number of affected regions, pain intensity and pain frequency (Figure 2); emotional exhaustion correlated with pain frequency and intensity but not with the number of affected regions, and depersonalisation correlated weakly with the number of affected regions and pain intensity. In contrast, QuickDASH showed no positive association with any symptom measure—a dissociation examined further in the Discussion. Neither age, BMI, occupational tenure, nor daily working hours correlated with any musculoskeletal outcome. The full correlation matrix is given in Table 4.

### 3.5. Low Versus High Symptom Burden (≤2 vs. ≥3 Affected Regions)

**Figure 3:**
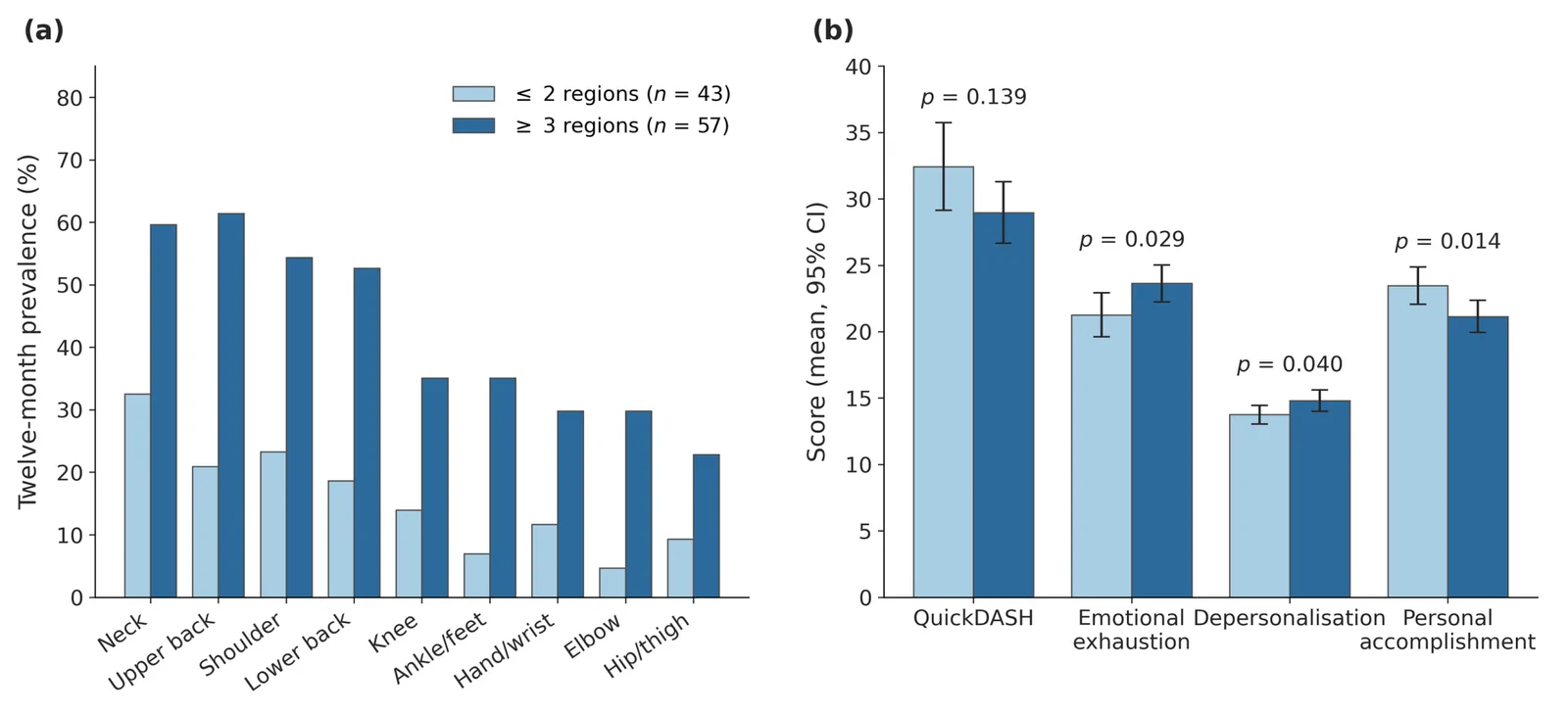
Low (≤2 regions) versus high (≥3 regions) symptom-burden groups: (**a**) regional twelve-month prevalence; (**b**) QuickDASH and Maslach Burnout Inventory subscale scores (bars: mean; error bars: 95% CI of the mean; *p* values from t or Mann–Whitney U tests as appropriate).

Forty-three participants (43.0%) had symptoms in two or fewer regions and 57 (57.0%) in three or more. The groups did not differ in age, BMI, daily working hours, sex, marital status, or education. High-burden workers had shorter occupational tenure and a consistently worse burnout profile—higher emotional exhaustion and depersonalisation and lower personal accomplishment—together with greater pain frequency and intensity, whereas QuickDASH did not differ between groups; group-wise estimates, test statistics and effect sizes are given in Table 5. In the Holm-corrected sensitivity analysis across the ten continuous comparisons, only pain intensity (adjusted *p* < 0.001) and pain frequency (adjusted *p* = 0.014) remained significant; the tenure and burnout differences (unadjusted *p* = 0.014–0.040) did not survive correction and should be regarded as exploratory. Regional symptoms were more prevalent in the high-burden group for every region; the association was strongest for the elbow (OR 8.71, *p* = 0.004), ankle/feet (OR 7.21, *p* = 0.002) and upper back (OR 6.01, *p* < 0.001), reached significance also for the neck, shoulder, lower back and knee, and did not reach significance for the hand/wrist (*p* = 0.054) or hip/thigh (*p* = 0.131) (Table 5, Figure 3).

**Table 5:** Comparison of low (≤2 affected regions) and high (≥3 affected regions) symptom-burden groups.

Variable	≤2 Regions (*n* = 43)	≥3 Regions (*n* = 57)	Test	*p*	Effect Size
Continuous variables — mean ± SD or median [IQR]					
Age (years)	37.0 [33.0–40.5]	33.0 [29.0–39.0]	U	0.064	0.19
Body mass index (kg/m^2^)	27.20 ± 4.31	27.43 ± 3.71	t	0.774	0.06
Occupational tenure (years)	12.0 [9.0–20.0]	10.0 [7.0–15.0]	U	0.035	0.21
Daily working time (h)	9.0 [9.0–10.0]	9.0 [9.0–11.0]	U	0.602	0.05
QuickDASH	30.45 [23.64–40.91]	26.51 [24.24–29.55]	U	0.139	0.15
Emotional exhaustion	21.26 ± 5.38	23.63 ± 5.24	t	0.029	0.45
Depersonalisation	13.0 [12.0–16.0]	15.0 [12.0–17.0]	U	0.040	0.21
Personal accomplishment	23.47 ± 4.59	21.14 ± 4.58	t	0.014	0.51
Pain frequency (0–4)	2.0 [1.0–2.0]	2.0 [2.0–3.0]	U	0.002	0.32
Pain intensity (0–5)	2.0 [1.0–3.0]	3.0 [3.0–4.0]	U	<0.001	0.40
Categorical variables—*n* (%)					
Male sex	29 (67.4)	34 (59.6)	χ^2^	0.555	–
Married	15 (34.9)	30 (52.6)	χ^2^	0.118	–
High education (≥high school)	25 (58.1)	36 (63.2)	χ^2^	0.762	–
Working ≥ 10 h/day	15 (34.9)	19 (33.3)	χ^2^	1.000	–

t, independent-samples *t*-test (effect size Cohen’s d); U, Mann–Whitney U test (effect size r = Z/√*n*); χ^2^, chi-square test with continuity correction. After Holm correction across the ten continuous-variable comparisons, only pain frequency and pain intensity remained significant (Section 3.5). –, not applicable.

### 3.6. Region-Level Comparisons

Burnout differences across regions were specific rather than diffuse. Elbow symptoms were accompanied by higher emotional exhaustion (*p* = 0.003) and lower personal accomplishment (*p* = 0.009); lower-back symptoms were accompanied by lower personal accomplishment (*p* = 0.035) and hand/wrist symptoms by higher depersonalisation (*p* = 0.009). QuickDASH did not differ by symptom status in any region (all *p* > 0.15). Participants working ≥ 10 h/day reported higher emotional exhaustion (median 24.0 vs. 21.5, *p* = 0.048) and a higher prevalence of elbow symptoms (32.4% vs. 12.1%, *p* = 0.030). Neck symptoms were more prevalent among those with high-school or university education (57.4% vs. 33.3%, *p* = 0.032). No outcome differed by sex or marital status (all *p* > 0.05), and Kruskal–Wallis tests across marital and educational categories were non-significant (all *p* ≥ 0.17). Given the number of region-level contrasts, these associations were not adjusted for multiplicity and should be regarded as exploratory.

### 3.7. Predictors of Upper-Limb Disability

In the primary multiple linear regression restricted to sociodemographic, occupational, and burnout variables, the full ten-predictor model did not explain significant variance in QuickDASH (R^2^ = 0.115, F = 1.15, *p* = 0.335; all VIF < 3.0) and no predictor reached significance. The secondary specification, including the pain characteristics, performed no better as a substantive model (R^2^ = 0.205, *p* = 0.050), the only surviving term after backward elimination being a paradoxical inverse association with pain frequency (B = −3.00, *p* = 0.004) whose residual diagnostics were unsatisfactory (Shapiro–Wilk *p* < 0.001). Upper-limb disability scores in this sample were thus not explained by demographic, occupational, burnout, or pain variables; this dissociation is addressed in the Discussion.

### 3.8. Logistic Regression for High Symptom Burden

**Figure 4:**
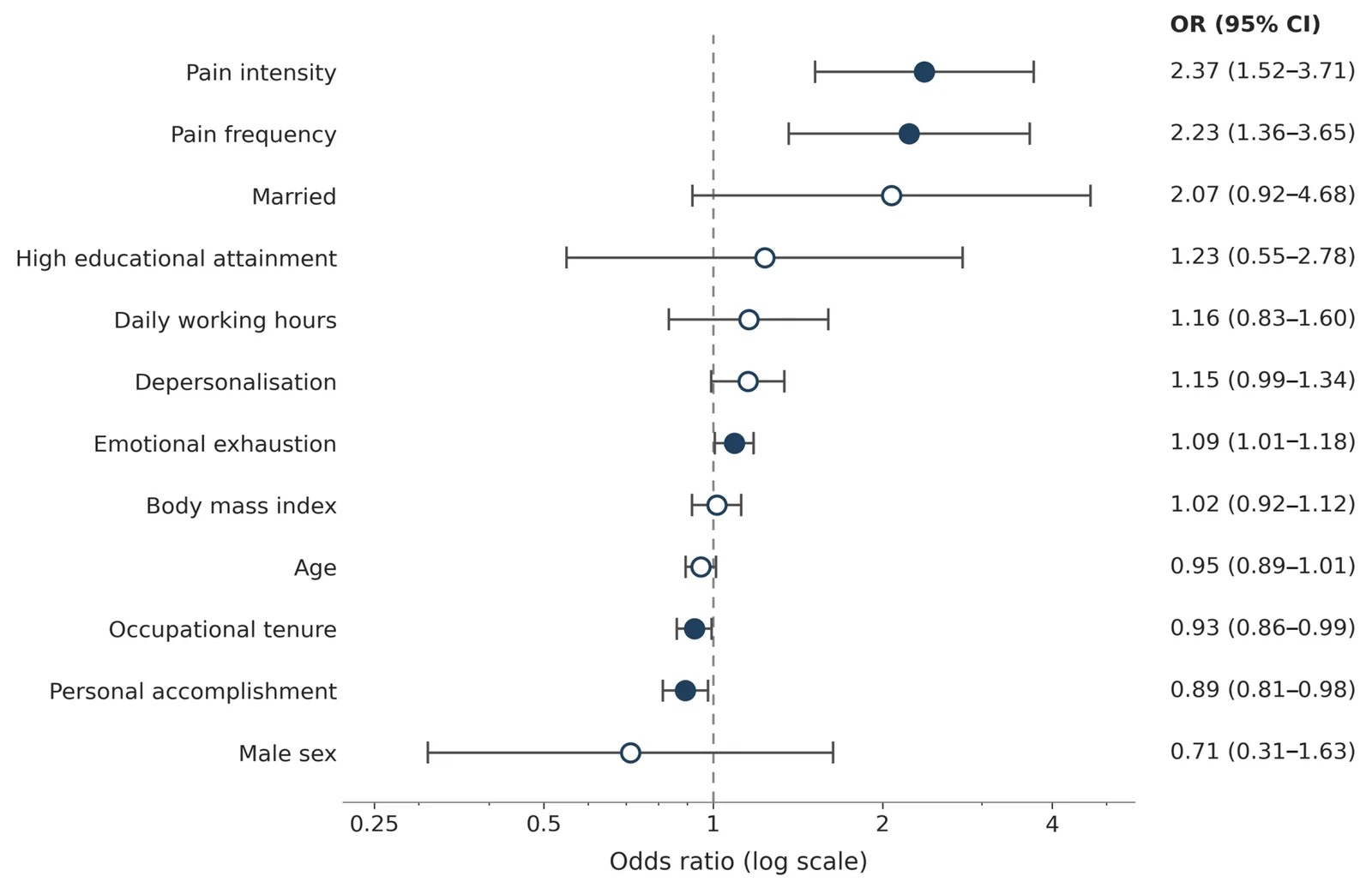
Univariable odds ratios (log scale) for high symptom burden (≥3 affected regions). Filled dark markers denote *p* < 0.05; the dashed line marks OR = 1; numeric estimates with 95% CIs are shown on the right.

In univariable analysis (Figure 4, Table 6), greater pain intensity and pain frequency, higher emotional exhaustion and shorter occupational tenure were associated with high symptom burden, and higher personal accomplishment was protective; age, depersonalisation and marital status met the *p* < 0.20 screening threshold only. Point estimates and confidence intervals are given in Table 6.

In the primary multivariable model (personal accomplishment, emotional exhaustion, occupational tenure, and depersonalisation), shorter tenure remained independently associated with high burden, while personal accomplishment attenuated to borderline significance; the model was well calibrated. In the secondary specification including pain characteristics, pain intensity was the only independent correlate. Full adjusted estimates and model statistics are given in Table 6.

### 3.9. Sensitivity Analyses: Continuous Symptom Burden

When the number of affected regions was modelled as a continuous count (Poisson regression with robust standard errors; primary covariate set), higher personal accomplishment remained independently associated with a lower symptom count (IRR 0.965 per point, 95% CI 0.943–0.987, *p* = 0.002), whereas occupational tenure (IRR 0.984 per year, 95% CI 0.967–1.001, *p* = 0.071) and depersonalisation (IRR 1.032, 95% CI 0.999–1.067, *p* = 0.060) attenuated to non-significance, and emotional exhaustion showed no independent association (*p* = 0.899). Univariable patterns mirrored the logistic results: pain intensity (IRR 1.28, 95% CI 1.16–1.41) and pain frequency (IRR 1.24, 1.10–1.40) were most strongly associated with the symptom count, and personal accomplishment was inversely associated (IRR 0.962, 0.945–0.980). These analyses indicate that the main conclusions do not depend on the dichotomisation of symptom burden, while suggesting that the association with personal accomplishment is more robust than the association with tenure.

## 4. Discussion

### 4.1. Principal Findings

This study provides a quantitative description of musculoskeletal symptoms, upper-limb disability and burnout in hairdressers working in women’s hairdressing salons in Istanbul, Turkey. Four findings were directly demonstrated. First, musculoskeletal trouble was close to universal: 95% of participants reported symptoms in at least one body region over the preceding twelve months, and more than half were symptomatic in three or more regions. Second, the anatomical distribution followed the biomechanical logic of the work, concentrating in the neck–shoulder–upper back complex (82%)—the regions loaded by sustained arm elevation and cervical flexion [6,7]—with the lower back close behind. Third, workers with a high multi-site burden were not those with longer exposure: high-burden workers had, if anything, shorter occupational tenure, and tenure was the only variable independently associated with burden in the primary logistic model, although this association attenuated when symptom burden was modelled as a continuous count. Fourth, all three burnout dimensions were worse in high-burden workers, with reduced personal accomplishment showing the most consistent pattern across correlational, group-based and sensitivity analyses. Two qualifications frame everything that follows: after Holm correction across the group comparisons, only the pain measures remained significant, so the tenure and burnout differences are exploratory; the cross-sectional design means that all findings are associations with no implication of causality or direction.

### 4.2. Plausible Mechanisms

The inverse association between occupational tenure and multi-site burden inverts the naïve dose–response expectation, and several mechanisms could plausibly produce it, none of which can be confirmed in this design. A healthy-worker survivor effect is the most parsimonious: hairdressers whose symptoms become limiting tend to leave the profession or reduce their hours, so long-tenured workers are a selected, more resilient group—a phenomenon documented in the Danish hairdressing cohort literature, where musculoskeletal pain predicted job change [6]. Skill acquisition offers a complementary explanation: experienced operators may have refined their working technique, tool handling, and pacing in ways that reduce exposure per client. The parallel age trend (high-burden workers were also non-significantly younger) is consistent with both accounts. Because the tenure association did not survive Holm correction and attenuated in the continuous-count analysis, these interpretations are offered as hypotheses rather than conclusions; if replicated prospectively, they would imply that prevention should target early-career hairdressers, in whom the burden appears concentrated, and work technique is still malleable.

A second set of mechanisms concerns the burnout findings. The most consistent correlational pattern belonged to reduced personal accomplishment, which tracked the number of affected regions and every pain measure, whereas emotional exhaustion attenuated in adjusted models and personal accomplishment was the only burnout dimension independently associated with the continuous symptom count. One speculative, hypothesis-generating interpretation is occupational rather than affective: in a craft occupation where earnings and professional identity depend on manual dexterity and stamina [18], pain that interferes with the quality or speed of work may erode the sense of professional efficacy more directly than it generates emotional depletion [17,34,37]. Direct occupation-specific evidence for this proposition in hairdressers is currently lacking, and we present it only as a candidate mechanism. The cross-sectional design cannot establish direction, and reciprocal causation—diminished efficacy reducing the motivation to maintain protective work technique—is equally consistent with the data.

A third mechanism question is raised by the dissociation between the QuickDASH and the symptom-based measures: upper-limb disability scores, although in the moderate range on average, showed no positive association with regional symptom reporting, pain characteristics, burnout, or the burden grouping. The QuickDASH asks about difficulty performing specific tasks, and experienced manual workers may maintain task performance despite symptoms through pacing, technique adaptation, and tool substitution—a form of functional compensation described in other craft occupations [6,15]. Response-shift and reporting styles may also differ between a task-based disability instrument and a body-region symptom checklist, particularly when questionnaires are completed in the workplace. Finally, measurement error in either instrument cannot be excluded. Whatever the mechanism, the finding cautions against relying on a single upper-limb disability score to characterise musculoskeletal burden in this workforce, and the QuickDASH results in this sample should be interpreted as descriptive only.

### 4.3. Findings in the Context of Previous Literature

The prevalence figures sit within, though generally toward the middle of, the wide ranges reported internationally for this occupation [6]. Our neck prevalence of 48% is comparable with the 53.4% reported in a large Ethiopian sample [10] and higher than the 26.3% reported among Indian metropolitan hairdressers [11], while our shoulder figure of 41% falls between the two. Our lower-back prevalence of 38% is somewhat lower than the roughly 53% reported in Ethiopia [10], and our lower-limb figures (knee 26%, ankle/feet 23%, hip/thigh 17%) are appreciably lower than the near-50% knee/foot estimate from India [11]. Differences in daily standing time, salon layout, chair adjustability and task mix plausibly explain part of this variation [6,12,13,14], as does our exclusion of workers holding a second job. The overall figure of 95% exceeds the 70% and 63% reported in Ethiopia and India [10,11]; two design features contribute—eligibility required at least five years in the profession, selecting for cumulative exposure, and the composite outcome counted trouble in any of nine regions. The figure should therefore be read as the prevalence of any self-reported musculoskeletal trouble among experienced hairdressers, not a clinical diagnosis rate.

The burnout findings extend the psychosocial literature on WMSDs [21,22,23,24,25] to a workforce in which burnout is seldom measured, despite pooled evidence of a substantial burnout prevalence among workers who treat or develop musculoskeletal conditions [38]. All three MBI dimensions differed between burden groups in the expected direction, in line with longitudinal evidence that psychosocial strain predicts subsequent musculoskeletal morbidity [21,22,39] and with the recovery-deficit model of WMSD development [6,25]. The association between long working days (≥10 h) and both emotional exhaustion and elbow symptoms echoes recent evidence linking long hours to multi-site pain [40]. Conversely, several variables often assumed to matter did not: neither age, BMI, nor daily working hours was associated with regional symptom burden in adjusted analyses, mirroring the null or inconsistent findings for these factors in other Turkish occupational samples [26,29,41,42]. The association between higher education and neck symptoms is difficult to interpret and may reflect differences in job role, reporting behaviour, or health literacy rather than exposure; it requires replication.

### 4.4. Hypotheses for Future Research and Implications for Practice

Pending confirmation in prospective studies, the results suggest—but do not establish—a two-track preventive approach. The physical track has independent experimental support: ergonomic redesign has measurable effects on WMSD pain [43], exercise programmes prevent neck pain [44] and improve work-related shoulder complaints [45], and combined ergonomic-plus-activity interventions outperform either alone [46,47]. Concretely for salons: adjustable cutting stools and chair heights that keep the shoulder below 60° of abduction, scheduled micro-breaks between clients, tool redesign to reduce sustained pinch grip, and progressive resistance exercise for the neck–shoulder girdle. The psychosocial track is less familiar: appointment scheduling that protects recovery time, skill-development and recognition mechanisms that sustain professional efficacy, and attention to the emotional demands of client-facing work [18,19,20]. Several testable hypotheses follow from our findings: reduced personal accomplishment precedes, rather than follows, the accumulation of multi-site symptoms; early-career hairdressers show the steepest symptom trajectories; employment position (salon owner versus employee) modifies the burnout-symptom association; and interventions that rebuild the worker’s sense of competent practice reduce symptom burden more than generic stress-reduction programmes. Prospective, multicentre cohorts with objective ergonomic exposure measurement (e.g., inclinometry or validated observational tools), repeated measurement of burnout and symptoms, and appropriate non-hairdressing comparison groups are the designs needed to test these hypotheses.

### 4.5. Strengths and Limitations

Strengths of this study include the use of internationally standardised instruments in their validated Turkish versions [29,32,33], complete data on all participants, the deliberate inclusion of both male and female operators—correcting a common mischaracterisation of this workforce—a pre-specified burden grouping, and an analytic strategy that reports models with and without conceptually overlapping predictors, constrains multivariable models by the events-per-variable rule [35], adjusts for multiple comparisons in sensitivity analyses, verifies the robustness of the burden grouping against a continuous specification, and reports discordant instrument findings transparently rather than selectively.

Several limitations must be acknowledged. First, the cross-sectional design precludes causal inference; the tenure and burnout associations are correlational, and the healthy-worker interpretation, while plausible, is unproven here. Second, all exposures and outcomes were self-reported, and no observational or instrumented ergonomic assessment (e.g., RULA, REBA, inclinometry) was performed, so exposure misclassification is possible and no postural dose–response could be examined. Third, the study was conducted in a single city: participants were recruited by voluntary response from salons affiliated with a single Chamber in Istanbul, so selection toward symptomatic workers cannot be excluded, and external validity to other Turkish cities, rural settings, unregistered salons and salons with different organisational structures is limited. Fourth, no formal a priori sample-size calculation was performed; with 100 participants the multivariable models were necessarily parsimonious, the confidence intervals around adjusted estimates are wide, and the precision of subgroup estimates is limited—the borderline adjusted association of personal accomplishment with burden in the logistic model (*p* = 0.100) and the attenuation of the tenure association in sensitivity analyses should be read in that light. Fifth, the questionnaire did not record employment position (salon owner/operator versus employee). Owners and employees plausibly differ in job control, income security, scheduling autonomy and client load—factors directly relevant to burnout—so residual confounding of the burnout–symptom associations by employment position cannot be excluded; this variable should be collected and modelled in future studies. Sixth, the SNQ records symptom presence dichotomously, without severity or duration within a region, and multiple comparisons were made; although a Holm-corrected sensitivity analysis is reported, the uncorrected exploratory findings may include false positives. Seventh, no clinical examination confirmed self-reported symptoms, so reported symptoms cannot be equated with diagnosed musculoskeletal disorders, and the absence of association between the QuickDASH and the symptom measures leaves the validity of the disability scores in this administration uncertain. Finally, the absence of a non-hairdressing comparison group means the prevalence estimates cannot be interpreted as an occupational excess over comparable non-hairdressing workers.

## 5. Conclusions

Musculoskeletal symptoms were close to universal among experienced hairdressers studied in women’s hairdressing salons in Istanbul, Turkey, affecting on average three body regions per worker and clustering in the neck–shoulder–upper back complex. Fifty-seven percent of workers reported symptoms in three or more regions; this high-burden group had a consistently worse burnout profile, greater pain frequency and intensity, and shorter occupational tenure—cross-sectional associations that are compatible with, but do not demonstrate, a healthy-worker survivor effect and a concentration of risk in early-career workers. Reduced personal accomplishment was the burnout dimension most consistently and robustly associated with physical symptom burden across the primary and sensitivity analyses. Within the limits of a single-city, cross-sectional design without a comparison group, these findings support piloting occupational health programmes that combine ergonomic redesign, scheduled recovery and targeted exercise with organisational measures that protect professional efficacy, reaching hairdressers early in their careers. Prospective, multicentre studies incorporating objective ergonomic exposure assessment, information on employment position, and appropriate non-hairdressing comparison groups are needed before causal or preventive conclusions can be drawn.

## Figures and Tables

**Table 1:** Sociodemographic and occupational characteristics of the participants (*n* = 100).

Characteristic	Mean ± SD	Median [IQR]/*n* (%)
Sex—male	–	63 (63.0)
Sex—female	–	37 (37.0)
Age (years)	35.27 ± 6.54	34.5 [30.0–40.0]
Height (cm)	163.55 ± 7.18	162.0 [158.0–169.3]
Body weight (kg)	73.20 ± 12.26	73.5 [63.0–82.0]
Body mass index (kg/m^2^)	27.33 ± 3.96	27.4 [23.5–30.4]
Occupational tenure (years)	12.55 ± 5.85	11.0 [8.0–17.0]
Daily working time (hours)	9.45 ± 1.24	9.0 [9.0–10.0]
Working ≥ 10 h/day	–	34 (34.0)
Marital status		
Single	–	48 (48.0)
Married	–	45 (45.0)
Divorced/widowed	–	7 (7.0)
Educational attainment		
Primary school	–	10 (10.0)
Secondary school	–	29 (29.0)
High school	–	46 (46.0)
University	–	15 (15.0)

SD, standard deviation; IQR, interquartile range. –, not applicable.

**Table 2:** Twelve-month prevalence of musculoskeletal symptoms by body region (*n* = 100).

Body Region	*n*	Prevalence (%)	95% CI
Neck	48	48.0	37.9–58.2
Upper back	44	44.0	34.1–54.3
Shoulder	41	41.0	31.3–51.3
Lower back	38	38.0	28.5–48.3
Knee	26	26.0	17.7–35.7
Ankle/feet	23	23.0	15.2–32.5
Hand/wrist	22	22.0	14.3–31.4
Elbow	19	19.0	11.8–28.1
Hip/thigh	17	17.0	10.2–25.8
Any region	95	95.0	88.7–98.4
Neck–shoulder–upper back complex	82	82.0	73.1–89.0
Any upper-limb region (shoulder, elbow or hand/wrist)	62	62.0	51.7–71.5
High symptom burden (≥3 regions)	57	57.0	46.7–66.9

CI, confidence interval (Clopper–Pearson exact method).

**Table 3:** Upper-limb disability, burnout and pain characteristics (*n* = 100).

Variable	Mean ± SD	Median [IQR]	Range
QuickDASH (0–100)	30.45 ± 9.74	27.12 [24.24–33.64]	16.67–58.56
MBI—emotional exhaustion (0–36)	22.61 ± 5.40	22.5 [19.8–26.0]	8–35
MBI—depersonalisation (0–20)	14.35 ± 2.79	14.0 [12.0–17.0]	6–20
MBI—personal accomplishment (0–32)	22.14 ± 4.71	22.5 [19.0–25.0]	9–32
MBI—summed score	59.10 ± 5.85	59.0 [55.0–63.0]	41–77
Number of affected regions (0–9)	2.78 ± 1.45	3.0 [2.0–4.0]	0–7
Pain frequency (0–4)	2.10 ± 0.93	2.0 [1.8–3.0]	0–4
Pain intensity (0–5)	2.63 ± 1.13	3.0 [2.0–3.0]	0–5

MBI, Maslach Burnout Inventory (Turkish adaptation, items scored 0–4); SD, standard deviation; IQR, interquartile range.

**Table 4:** Spearman rank correlations between musculoskeletal outcomes and burnout, pain, and occupational variables (*n* = 100).

Variable	QuickDASH	No. of Regions	Pain Frequency	Pain Intensity
Emotional exhaustion	−0.022	0.187	0.324 ^†^	0.329 ^†^
Depersonalisation	−0.149	0.236 *	0.143	0.204 *
Personal accomplishment	−0.085	−0.295 ^†^	−0.211 *	−0.325 ^†^
Number of affected regions	−0.066	–	0.311†	0.465 ^†^
Pain frequency	−0.261 ^†^	0.311 ^†^	–	0.436 ^†^
Pain intensity	−0.161	0.465 ^†^	0.436 ^†^	–
Age	−0.161	−0.132	0.037	0.021
Body mass index	0.066	0.082	−0.026	−0.043
Occupational tenure	−0.163	−0.142	0.052	0.052
Daily working hours	0.031	0.067	−0.054	0.128

Values are Spearman’s ρ. * *p* < 0.05; ^†^ *p* < 0.01.

**Table 6:** Univariable and multivariable logistic regression for high symptom burden (≥ 3 affected regions; 57 events/43 non-events, *n* = 100).

Predictor	Crude OR (95% CI)	*p*	aOR (95% CI)—Model A	*p* (A)	aOR (95% CI)—Model B	*p* (B)
Pain intensity	2.37 (1.52–3.71)	<0.001	not entered	–	1.90 (1.14–3.15)	0.013
Pain frequency	2.23 (1.36–3.65)	0.001	not entered	–	1.51 (0.84–2.70)	0.167
Personal accomplishment	0.89 (0.81–0.98)	0.017	0.91 (0.82–1.02)	0.100	0.95 (0.85–1.06)	0.356
Emotional exhaustion	1.09 (1.01–1.18)	0.033	1.04 (0.95–1.14)	0.386	1.01 (0.91–1.11)	0.846
Occupational tenure	0.93 (0.86–0.99)	0.034	0.92 (0.85–0.99)	0.024	not entered	–
Depersonalisation	1.15 (0.99–1.34)	0.062	1.14 (0.97–1.34)	0.108	not entered	–
Married	2.07 (0.92–4.68)	0.079	not entered	–	not entered	–
Age	0.95 (0.89–1.01)	0.115	not entered	–	not entered	–
Daily working hours	1.16 (0.83–1.60)	0.384	–	–	–	–
Male sex	0.71 (0.31–1.63)	0.425	–	–	–	–
High educational attainment	1.23 (0.55–2.78)	0.611	–	–	–	–
Body mass index	1.02 (0.92–1.12)	0.771	–	–	–	–

OR, odds ratio; aOR, adjusted odds ratio; CI, confidence interval. Candidates (univariable *p* < 0.20) were entered in ascending order of *p* value up to four covariates (EPV ≥ 10). Model A—primary specification (sociodemographic, occupational and burnout variables): Nagelkerke R^2^ = 0.191, Hosmer–Lemeshow *p* = 0.482, AUC = 0.714, LR test *p* = 0.004. Model B—secondary specification (pain characteristics eligible): Nagelkerke R^2^ = 0.266, Hosmer–Lemeshow *p* = 0.894, AUC = 0.754, LR test *p* < 0.001. –, not applicable.

## Data Availability

The data presented in this study are available on request from the corresponding author due to privacy and ethical restrictions: the informed consent signed by the participants and the ethics committee approval do not cover public deposition of individual-level data. A de-identified minimal dataset can be shared with the editorial office or reviewers for verification purposes upon request.
